# Progress of the Medical Sciences

**Published:** 1891-03

**Authors:** 


### Progress of the Medical Sciences.

### MEDICINE.

Although it is impossible at present with the data at our disposal, and in the short interval of time that has elapsed since Koch gave his discovery to the world, to form an accurate judgment of its ultimate value, there has been a certain amount of experience accumulated which is of value to guide us in the further use of the remedy and to indicate the ways in which, by further research, we may hope that the advantages attending its use may be increased and the drawbacks lessened. The exact constitution of this so-called "tuberculin" is uncertain, and must remain so until Koch publishes in greater detail the mode of preparation. He states that it is a derivative of albuminous bodies, and is in close relation to them, but that it does not belong to the group of bodies known as toxalbumins. As to its mode of action, it contains a certain amount of a necrosis-producing substance formed during the life-activity of the tubercle bacillus; and this substance, injected in minute quantity, excites in tuberculous subjects a more or less extensive necrosis of cells wherever the tubercle-bacilli vegetate, and have already impregnated their surroundings with the necrosis-producing body. Accompanying these local changes are general symptoms affecting the entire organism, consisting of fever and the complex of symptoms known as the "reaction."[1] This reaction is in fact an acute specific fever of short duration excited by the injection of tuberculin.

As for the local effect, it falls only indirectly on the bacillus itself by interfering with, or altogether preventing, its nutrition. Briefly, in a focus of tubercle a central area of necrosis is produced, surrounded by a zone of intense hyperæmia. In the case of ulcers (larynx and intestine), instead of a greyish base with swollen and red edges, they show a surface covered with recent very vascular granulations. The tuberculous are thus transformed into simple ulcers.[2] In the lung it seems

[1] *Brit. Med. Journ.*, Jan. 17, 1891.

[2] Jürgens, *Wiener med. Woch.*, Jan. 3, 1891.

likely that, if these are extensively diseased, caseous hepatisation may occur throughout whole lobes. The grave question has been raised by Prof. Virchow as to whether there was not a fresh development of miliary or submiliary tubercles in distant parts in some patients who had died whilst under treatment. His latest views on the effect of the remedy are, that up to the present we have no evidence that the bacilli as such are killed and re-absorbed; that the tubercle, as well as the irritable tissue, the inflammatory tissue around it, is brought to a speedy destruction by the treatment, thus favouring the possibility of a comparatively rapid healing process. On the other hand, there is no evidence that indurative processes are favourably influenced, or that encapsulation is favoured; rather, suspicion sometimes arises that the remedy mobilises encapsuled masses, brings them into movement, and thus transforms a focus of disease, which might possibly have continued to be harmless, into an actual danger for the patient.[1]

So far as present experience goes, the defects of the remedy arise in some cases from the violence of the local, in others of the general, reaction. In the first place, in deep-seated organs the necrosed material, if in any large amount, cannot at once be discharged, and there is a quantity of *detritus* which can only be removed by a long and exhausting process of suppuration. In certain situations, again, the intensity of the hyperæmia of the tissues surrounding the tubercles is a source of danger, as in the case of the boy reported in Virchow's first paper,[2] in which an extraordinary degree of hyperæmia of the pia mater was excited by the injections. In the larynx a condition resembling œdema glottidis may supervene; or again in the intestine, in the course of the secondary changes taking place around a tuberculous ulcer, the necrotic process may extend to the serous coat and result in perforation, as in a case mentioned by Dr. B. Fraenkel. On the other hand, danger may arise from the intensity of the general reaction, which seems to vary in a way which cannot be foreseen, and is at present unexplained. In one frequently quoted case, that of a girl *æt.* 17, suffering from lupus, death followed 36 hours after an injection of ·002 gr.

The fever may be attended by albuminuria, tumefaction of the spleen, and even myocarditis or hæmaturia. A severe general reaction is not necessarily accompanied by a corresponding intensity of the local effects; and this inadequacy of

[1] *Brit. Med. Journ.*, Feb. 21, 1891, p. 434.
[2] *Brit. Med. Journ.*, Jan. 17, 1891.

the local effect, whilst the general disturbance is severe, must be accounted one of the defects of the remedy.

Sometimes the obstacle to success lies in another direction—namely, the establishment of tolerance and consequent exhaustion of the action of the remedy before the cure is effected: sometimes even, as the results of autopsies have shown, the necrotic process does not occur, even large tubercles remaining refractory. There appears to be no doubt that the injections may occasionally have a disastrous effect by rousing into fresh activity old quiescent lesions, whose presence had clinically been previously unsuspected. The statement of Liebmann that he had found bacilli in the blood during the reaction period has not been confirmed by other observers. The many observations that have been made on the sputum in pulmonary phthisis show that at first both the quantity of sputum and the number of bacilli it contains are increased. The bacilli may be considerably altered, the rods being broken down and irregular in shape, whilst they often do not retain the stain in the presence of acids so well as usual, and may show a tendency to occur in groups or masses. In a considerable proportion of cases the amount of elastic tissue is increased. These changes connote the active disintegration and discharge of the tuberculous pulmonary tissue. In those cases that do well, the amount of sputum and the number of bacilli subsequently diminish, and in a few the bacilli have altogether disappeared and remained absent, whilst expectoration and cough have ceased.

With regard to the action of tuberculin in pulmonary phthisis, the time has not yet come to speak definitely; but it may be said that on the whole Koch's statements that tuberculin has a specific action on tuberculous tissue, that this action would be available only in cases in the early stage and not in those attended with extensive changes in the lung, have been realised by clinical experience. The effects, however, even in early cases, have, so far, not been so beneficial as he anticipated. It must be remembered that we cannot argue directly from the results of laboratory experiments on animals, the subjects of artificial tuberculosis, to pulmonary tuberculosis in man. Tuberculin, while destroying existing foci of disease, does not appear to be able to protect against future attacks. As Dr. Nixon points out, in artificial tuberculosis the products of the bacillary culture are applied to an acute infectious disease whose course is not affected by predisposing causes. In phthisis, on the other hand, the hereditary taint, the defective assimilation, and diminished vital power of resisting morbid influences must be taken into account.[1] On exposure to fresh sources of infection, such patients may, after cure, be again liable to contract the disease; and, moreover, if after the treatment some bacilli remained behind undestroyed—a by no means improbable case—these might be roused into fresh activity if the patient were subsequently placed under conditions favourable to their development. These are two of the limitations to complete success. There is another danger from the tendency of the necrosing tissue from the upper parts of the lung, if not rapidly expectorated, to gravitate into the lower lobes and thus infect parts of the lung not previously attacked. That this may occur is shown by the reports of cases in which signs of mischief were first observed over the bases of the lungs after injection, and by the extensive caseous hepatisation found *post mortem* by Virchow.

The best results in phthisis may be anticipated in early cases when the consolidated areas are just beginning to soften. Cases of advancing phthisis with formation of cavities do not do so well. As a certain percentage of moderately severe cases grow worse under the treatment, patients of this class should be warned of the dangers to which it may possibly expose them.[2]

In reading the reports one sees that hæmoptysis has occurred with some frequency after the injections, as would be expected, knowing the inflammatory condition of the tissues surrounding the tubercular foci, produced by the action of the remedy. Prof. Fraenkel states, and analysis of the reported cases from all sources appears to confirm this statement, that improvement in the general condition of the patient results in from 40 to 50 per cent.—taking only those cases that have been a sufficient time under treatment and are in an early stage of the disease,—but that it is doubtful whether this improvement is greater than follows in the same class of cases under the ordinary tonic, dietetic, and antiseptic regimen.

Careful selection of patients is essential; and since the intensity of the general reaction is most uncertain and cannot be foreseen, varying apparently according to individual idiosyncrasy, it is well to begin with a very small dose—½ to 1 milligramme. The treatment should not be attempted in advanced or bad cases of phthisis; and other contra-indications are, the presence of symptoms pointing to intra-cranial tuberculosis, extensive pleuritic effusion, nephritis, and tubercular disease of the intestines. For the rest, there seems to

[1] *Brit. Med. Journ.*, Feb. 14, 1891, p. 357.

[2] Prof. A. Fraenkel, *Brit. Med. Journ.*, Feb. 21, 1891, p. 434.

be little risk if the cases are judiciously selected, the initial dose employed a small one, its effect carefully watched, and the dose gradually increased. The improvement that has resulted in many patients is certainly very remarkable and of good augury for the future.

The results in the treatment of lupus are not so encouraging as at first reported, good authorities having stated that tuberculin does not attack the true lupus tissue, and that in spite of great improvement at first, recrudescence of the disease may take place later. Prof. Koehler thinks that when the lupus is not quite superficial the throwing off of the necrosed tissue is prevented by the cutis, which remains intact; the injections, therefore, require following up by scraping with a sharp spoon. He gives one case—the most successful hitherto—in which the lupus tissue was superficial. After treatment by injection only, the lupus has completely disappeared, and the diseased areas are covered by healthy skin with excretory ducts and a growth of lanugo. The combination of injections with scraping seems to promise better results in a shorter time than have hitherto been obtained by other remedies.

Whether tuberculin will come into any extensive use as a diagnostic agent in obscure and difficult cases seems doubtful, in view of the severe effects that sometimes result from a dose of sufficient strength for diagnostic purposes. Whatever may be the immediate results of the first trial of "tuberculin," there can be only one opinion as to the great importance of Koch's discovery, which promises to open a new era in medicine, and to pave the way for great advances in a new line of treatment. We may hope that by the light of further experience the deleterious effects of the remedy will be attenuated, if not altogether eliminated, and the beneficial effects intensified and rendered more potent; possibly at no distant date we may be led to the discovery of some substance that will act directly upon the bacilli themselves.

J. Michell Clarke.

### SURGERY.

A surgical operation is, after all, a therapeutic measure, and we cannot too strongly or too often insist on this fact.

Some surgeons seem to regard an operation merely as an anatomical exercise to be pursued after set methods, and described in skeleton in parallel columns. We mistrust operations after set methods. It may be taken for granted that any list of a hundred examples of the radical cure of hernia, after the method of Snooks, contains at least ten examples of bad surgery, and twenty more of a surgery that is not the very best. And we have no love for lists of operations arranged as statistics, in which one column contains the initials of the patient, another the age, another the date of operation, another the practitioner who sent the patient, and the last column of all one or other of the letters "R." and "D." From such a table containing the items proving that Mr. Stubbs has five hundred times removed the vermiform appendix, we can infer nothing beyond the probability that at least one hundred of these operations were pure mutilations. It is as if a man provided us with five hundred examples of the administration of one ounce of castor oil, the final results being tabulated as "S." success, "F." failure; which being fully interpreted, means that the bowels acted or failed to act. The effects of the drug on the disease are quietly ignored; but we are supposed to infer that the patient is cured.

Some of us refuse to make any such inference. But our enthusiastic table-compilers care nothing for this; for Mr. Snooks is now the most experienced of rupture-curers, and Mr. Stubbs is the leading appendix-vermiform-ectomist of the day. Patients flock to them at their special hospitals—for nearly all such surgeons are specialists—unbeknown to their family doctors. If the poor man with ingrowing toe-nail, who slips away to the "Ingrowing Toe-nail Hospital," ventures to grumble somewhat that the cure consists in complete removal of the big toe, he is solaced by the true statements that he has suffered much and long at the hands of many doctors, and that he is now cured. Yes; but at the expense of the big toe!

There has been a recrudescence of the discussions about removal of the uterine appendages. A young surgeon of unformed reputation and small experience presents a series of cases of the operation larger than surgeons of enormous experience and world-wide reputation could present in the same time. This is the signal for a cry of amazement from many, who want to know where all this mass of disease justifying these operations came from. Some boldly affirm that it did not exist; others, that there was an overdoing of therapeutics, like our amputation of the big toe for ingrowing nail; while the ultra-conservatives affirm that the tedious and unsatisfactory recovery of these cases renders the operation unjustifiable, except in cases where life is undeniably in danger. It is probably the fact that among the most capable and experienced men the conservative view is gaining ground, and only the most severe cases with palpable and extensive disease are submitted to operation. And from such men, time after time, comes an emphatic protest against the publication of such cases as cured merely because they have recovered from the surgical operation. We add our protest to the others, and with it a warning that if more caution is not observed, an operation which is for many suffering women a beneficent therapeutic measure will become totally discredited.

Koch's treatment is now in full swing all over England. Some of us have been experimenting with it for more than three months, and might reasonably have expected to be able to present some definite results. But we have no definite therapeutic results to describe. The time when a case can be said to be cured has not arrived; but we have had cases of recurrence after supposed cure already reported. Mr. Hutchinson, in his address before the post-graduate students, summarises in a conspicuously fair and judicial manner the general feeling with regard to the value of the fluid in lupus—namely, that it is certainly not superior to other methods of treatment, and perhaps inferior to some. And even in this position of modified excellence it is a treatment, in the most careful hands, that is by no means free from danger. The discussions now going on in Berlin help to exhibit the special modes in which the fluid conduces to death; we are not anxious to be presented with such opportunities of *post mortem* study. The discovery by several competent observers of free bacilli in the blood of injected patients suggests an alarming possibility which we do not care to contemplate. The recrudescence of acute tuberculosis after a few months in injected patients would be a terrible commentary on the failure of the most gigantic experiment that has ever been performed on human beings.

It is a thousand pities that Koch's hands were forced in the way that they were forced. Left to his own bent for a year or two more, he, who is most competent to experiment and observe on the matter, might have been able to give us some final and conclusive utterances. But the experiment has gone from the control of the trained expert to the trials of the untrained practitioner, and we cannot say where or when it will end. Koch is on the right path—*rem acu tetigit*, literally as well as metaphorically—it is the fault of this bustling and advertising age that the calm scientist has not been permitted quietly to finish his work.

In the meantime several skilled observers are doing good work at the same subject: Metschnikoff in particular, working in Pasteur's laboratory, has carried out some most valuable researches on the production of immunity to infection by specific micro-organisms, and on phagocytosis. Ruffer has supplemented and extended Metschnikoff's observations by close and painstaking researches on the actual process of phagocytosis during the progress of inflammation.

The process of phagocytosis, or the destruction of micro-organisms by living cells in the body, is one of far-reaching importance to the surgeon. One proof of its existence, in concomitant variation of cause and effect, is suggested if not demonstrated by the fact that the more malignant the micro-organism the more rarely is it found within the phagocyte; and *vice versâ*, the slower the blood-poisoning the more abundantly are the cells found in phagocytes. On these data a theory of immunity has been founded which simply depends on the abundantly proved theory of memory or habit of tissues to react with increasing facility to stimuli. Immunity or refractiveness to inoculation would simply mean that the phagocytes have acquired from practice the habit of destroying bacteria. In this view, the giant cells of tubercle are simply huge multi-nuclear phagocytes. We anxiously await further accounts of these interesting observations.

In this connection it is interesting to note that, according to Fehleisen, quoted by Woodhead in a philosophic article in the last volume of Laboratory Reports, just published by the Edinburgh College of Physicians, erysipelas was in past times undoubtedly used as a curative agent. On lupus in particular the effects of erysipelas as a curative agent have recently not only been noted, but put to a practical test. Woodhead's own experiments show that immunity to one disease might be produced by injection of the germs of another; and, what is more remarkable, by the sterilised fluids got from artificial cultures of these germs. Some observers indeed maintain that the sterilised cultures are more powerful agents in producing immunity than the germs themselves. On the other hand, it has been found that the importation of another microbe to the infection, instead of counteracting the effect of the first, may increase its virulence. Any conclusions drawn from similarity of structure in the micro-organism are as yet impossible; the only fact thus far quoted in this direction is the suggestive one that the bacilli of tuberculosis and of leprosy, phylogenetically related as they are, react similarly to Koch's lymph.

Koch's treatment is not on the lines of these investigations.

It seeks by a deliberate increase of the tubercular toxines to aggravate the local inflammation, if possible, to the extent of necrosis. This is evacuation or elimination, rather than immunity. Time has yet to show in which direction the best practical results are to be attained.

Is there any one *best* mode of performing colotomy?

At present the leaning is towards inguinal colotomy, or, as it might with more propriety be called, cœlio-colotomy. Reports of different series of cases operated upon, according to their special methods, by Harrison Cripps, Allingham, Rose, Ball, and others have shown excellent results as regards both immediate mortality and remote effects. The advantages of cœlio-colotomy have been frequently urged, and are now fully appreciated; but it has not all the advantages on its side. We have met with a condition where malignant stricture, low down in the rectum, was associated with enormous distension of the whole colon with liquid fæces and aggravated symptoms of intestinal obstruction, with great weakness of the patient; here lumbar colotomy seemed to be the best operation. Immediate opening was essential; profuse intestinal discharges had to be provided for; and the excessive distension of the gut would severely test any method of fixation in the abdominal wound. Under these and similar circumstances, the old lumbar method has manifest advantages. But, as a general rule, the abdominal method will be the operation of election.

A study of the various modes of performing cœlio-colotomy enables us to eliminate defects and pick out virtues. Most of the defects are errors on the side of excessive caution. A few of these may be indicated. All methods of elaborate suturing are unnecessary. Thus, it is not necessary to suture parietal peritoneum to skin; bowel unites to the open incision just as quickly as to parietal peritoneum, and it is fixed on a firmer foundation without mobile fat under it. Nor is it essential to place more than a very few sutures between the bowel and the parietal opening. Indeed, in what is probably the best of all methods—namely, that of Maydl as modified by Reclus—no sutures at all are essential. The bowel simply rests on a skewer passed through the mesentery and laid on the abdominal walls. The supporting suture of Allingham was a step in this direction; the same principle has been utilised by Rose, only that the supporting sutures are (as the writer suggested two years ago) carried well outwards through the parietal tissues. Nothing keeps the bowel in the wound so efficiently as this underlying bridge. The advantages of the skewer, protected with antiseptic gauze or lint, are, that its breadth prevents the chance of ulceration of the mobile gut, and that it is applied with the greatest possible ease and speed. It is no small advantage in favour of such an operation as colotomy, that it can be performed quickly. The operation of Reclus can easily be completed in five minutes. It provides an excellent anus, with a suitable spur; and, if we deem it advisable, it is quite easy subsequently to close the lower opening after complete division of the gut.

Resection of a portion of intestine so as to prevent intestinal prolapse should also be unnecessary. If the upper bowel is pulled downwards, and the wound is not too large, there should be no prolapse. To cut the bowel through, and close up and return the lower portion, may in one case out of ten or twenty end in disaster. This would occur if there resulted complete closure at the stricture, when the discharges would be forced upwards into the closed gut. And the advantages of so returning the bowel are so slight, that this risk is not counterbalanced.

Also, removal of protruding pieces of gut need rarely be carried out. The portion of intestinal wall around the opening shrivels up and becomes covered with skin; in fact, assimilates the artificial to the natural anus by a sort of inversion of skin rather than eversion of mucous membrane, thus preventing escape of mucus and irritation of exposed mucous membrane. The delay of a day or two in opening may, in all cases except those with enormous colic distension, be secured by removing gas and fluid fæces through a large aspirator needle, repeated as often as seems desirable. The small opening made by the needle may easily be closed up by a Lembert's suture.

J. GREIG SMITH.

### OPHTHALMOLOGY.

Edward Jackson of Philadelphia, at the Ninth International Congress, held at Washington, brought forward a proposition that prisms should be numbered according to their *power of deflecting light* in its passage through them, and showed that the present method of designating them according to the number of degrees of the *reflecting angle* bears no accurate ratio to their power, on account of the varying and uncertain refractive index of the glass used in their manufacture. The author, with Landolt and Swan

Burnett, were appointed a Committee to investigate and report upon the matter.

Since this time much has been written on the subject. The general principle that prisms should be designated *according to their power*, and not according to their shape, has been very unanimously accepted; but the question still remains, What is the best way of numbering prisms?

Two authorities have suggested that the *angle* through which the ray is deflected should be measured:

(1) Jackson originally proposed to designate the prism by the *number of degrees* it deflected light passing through it in the direction of minimum deviation. Landolt also supports this view. He says: "We appreciate and express the excursions of the eyes as well as their anomalies in *angular degrees*. The field of fixation, the angles of strabismus, are objectively measured by instruments which are divided into *degrees*." As the deviation produced by a glass prism may be regarded as equal to half the angle of figure of the prism, an easy calculation on the present marking could be made.

(2) Dennett wishes to use the "Radian" (the angle whose arc is equal to the radius 57.295°) as a basis for the new system, making the standard prism one which deflects light one hundredth part of a radian; and this unit prism he proposes to call a "Centrad" = .57°. A prism causing deflection through $x$ times this angle, would be designated $x$ centrads.

(3) Prentice, on the other hand, finds it more practicable to measure the *base* of the angle through which the ray of light is deflected. He takes the linear measurement of deflection on a plane surface, situated at the distance of a metre from the prism; and he fixes his unit, which he calls a "Prism dioptre," at one centimetre on the metre plane. Swan Burnett writes strongly in support of adopting Prentice's suggestion. He says: "While it is exceedingly difficult, and only by aid of complicated apparatus, to measure the minimum angle of deviation, or the centrad, the amount of deflection on a plane surface is most easily determined." We have only to place a centimetre measure at right angles to the line of vision, at a metre distance, and, looking through the edge of the prism, read off the amount of displacement on the scale in centimetres of a vertical line, as seen above and below the edge of the prism tested: No. 1 gives a deflection of 1 c.m.; No. 4, of 4 c.m.; $\frac{1}{2}$, of 50 m.m.; and the ratio of displacement being 1 to 100 (1 c.m. to 1 metre), the calculation can be as easily made, taking any multiple of the metre as the distance of the plane. The decentring of lenses is very simple on this method; 1 c.m. of decentration gives as many prism dioptres as the lens contains dioptres of refraction.

Burnett adds that the present numbering of prisms happens to correspond very nearly to the numbers in prism dioptres, and that the stock of prisms now in use could be still utilised under the new nomenclature; that the metric standard is thus maintained, and he quotes a firm of manufacturing opticians as expressing themselves strongly in favour of the metric system of grinding, and of the practical value of Prentice's prismometer, which measures to $\frac{1}{100}$ of a prism dioptre; that the value of the metre angle of an individual is easily expressed in prism dioptres according to his interpupillary distance.

Jackson points out that the methods of Dennett and Prentice are almost identical; the "centrad" is $\frac{1}{100}$ of the radius measured on the arc; the "prism dioptre" is the $\frac{1}{100}$ of the radius measured on the tangent; and that for practical purposes as applied to prisms used in ophthalmology, they may be regarded as identical, and show, at any rate, less inaccuracy between one another than the sets of trial prisms now in use. Both prism dioptre and centrad bear an extremely simple relation to the metre angle—the radius of which is 1 metre, and the tangent half the distance between the pupils. Each centimetre of half the interpupillary distance gives one centrad of angular deviation. The metre angle of 6 c.m. interpupillary distance (rather below the normal) is 3 centrads; it also equals, very nearly, 3 prism dioptres. The difference between the arc (the centrad), the tangent (the prism dioptre), and the sine (the metre angle) is practically the same for a prism of 5° or under; for a prism of 10° the arc = .17451; the tangent = .17633; the sine = .17365.

There is, however, no real analogy between the metre lens and the metre prism; and no reason exists between them for the choice of 1 c.m. as the standard of deflection, whether it be measured on the plane or on the arc. The centrad .57° is nearly equivalent to half a degree; and it is difficult to see what useful purpose is served by introducing a word which suggests no analogy in terms with the purposes to which it would be applied, because all our ideas for the use of prisms are, and will continue to be, measured by the degree. The writer is of opinion that ophthalmological prisms should be marked in degrees and minutes according to their minimum angle of deviation. The work done by the prism would thus be accurately stated in the same terms as are used for the conditions it is intended to control. The comparison of this marking with the metre angle is readily made, and a convenient series of standard strengths would find their way into the trial cases; or else a standard prism of 15 minutes might be taken to be called a "quarter," and its multiple $x$ be marked $\frac{x}{4}$, the essential point being to retain the degree as the basis of calculation.

The centrad has been somewhat hastily adopted as a unit of strength for prisms by the American Ophthalmological Society—July, 1890. At the International Medical Congress little interest was taken in the matter; but it is quite certain that it will not be allowed to rest.

Consideration must be more and more directed to this question of prisms, as the subject of muscular insufficiency and latent deviation of the eyes is just now receiving so much attention, particularly in America.

The terms suggested by Stevens, according to the various tendencies of the visual axes, Hetero-phoria, Eso-, Exo-, Hyper-phoria seem to be coming into more general use, and the large amount of labour he has given to this subject has at any rate called attention to it, however much its importance has been exaggerated.

Thus it is even claimed, (1) That faulty relations of the eye muscles, through eye-strain, cause epilepsy, chorea, and similar disorders. (2) That these faulty conditions may be rectified, and these disorders cured by graduated tenotomies. The Report of the Committee of the New York Neurological Society, appointed to investigate the subject of treatment recommended by Dr. Stevens by lenses, prisms, graduated tenotomy, or advancement of ocular muscles for neuroses, of which epilepsy and chronic chorea were taken as types, resulted in an expression of opinion very unfavourable to Dr. Stevens's views; and during the last few months many have stoutly denied the very existence of muscular insufficiencies as a cause of discomfort, and have denied that prisms or operations are of any use in any form of asthenopia.

Not a few agree with Stevens that all sorts of reflex nerve troubles depend alone on muscular defects; others err in the opposite direction. Thus, Roosa disputes the whole of Stevens' doctrine regarding muscular asthenopia, and considers that all cases in which the symptoms thus named exist, they are due to errors of refraction. If insufficiency of the recti is clearly shown to exist, it is due to the refractive error present. Many of the cases are astigmatic, and Javal, since the use of his ophthalmometer, now very seldom prescribes prisms, and Roosa has entirely given them up.

Very much the same views were expressed by many of the authorities at the Berlin Congress. Landolt, however, still holds that asthenopia from muscular insufficiency really exists, and the author of this report cannot but agree with him, both upon the perusal of published cases and from personal observation; although, no doubt, they are not of such common occurrence as some observers indicate.

Woodward says that there are numbers of cases in which the error of refraction has been properly corrected, but the disappearance of the error in the ocular muscles has not ensued as a consequence. The correction of the anomalies of the ocular muscles may be made by resorting to either prisms or operative interference: prisms, as a rule, are unsatisfactory in a certain proportion of cases, in some they serve our purpose very well. The modern operative methods are based upon a much more thorough understanding of the visual apparatus than formerly obtained, and when performed by a competent person are eminently satisfactory. (This criticism, on the other hand, is perhaps rather too flattering.)

Noyes has found prisms of much service in cases of muscular asthenopia, the large majority of which were due to insufficiency of the external recti. Most of his patients followed some occupation requiring persistent eye-work, and the majority had good general health. The principal symptoms were, headache on waking in the morning, pain in the eyes, blurred vision, spasm of accommodation, difficulty in keeping the eyes fixed on one point, or in following a moving object, and vertigo. In a very large number the refraction is said to have been emmetropic. Three-fourths of the cases were quite relieved by use of prisms, of a strength, as a rule, not more than three degrees, in combination. In three cases tenotomy was done.

Dr. Allen Starr, in considering "peripheral irritation and nervous phenomena," said that in testing the power of ocular muscles he had observed under the same tests, in the same individual, at different times, considerable variations, depending upon the patient's general condition. In epilepsy there was often evident variation in equilibrium of the ocular muscles, before and after the attack. In neurasthenia and other functional diseases the ocular muscles might at times show a relative weakness; and by directing a patient's attention to an organ, he became conscious of sensations that seemed abnormal, though they might be perfectly natural. But out of three thousand cases of nervous disease, he could cite only one case of true nervous manifestation undoubtedly caused by eye-strain.

Hewetson claims to have cured an epilepsy by correcting the refractive error, and says that myopic astigmatism has caused *petit mal.*

Eales says that headaches due to ocular causes, however general and diffused they may become after they have set in, invariably commence in one or both of the eyes themselves, or more commonly over one or both eyebrows, or the forehead, or over one or both temples, or over the root of the nose; and that the great majority of such headaches are due to ocular conditions. He thinks that eight or ten hours a day is the maximum time, not continuous, that the effort of accommodation can be maintained in a healthy eye without discomfort.

Galezowski advocated, in retinal detachment, tapping off the subretinal fluid, and injecting iodine between the retina and the choroid.

Schoeler has reported twenty-eight cases of detachment, in which he has injected one or two drops of tincture of iodine in front of the detachment, so as to separate it from the overlying vitreous, and to encourage plastic adhesion between retina and choroid. One-third of the cases were stated to be cured, fully and permanently; in another third temporary benefit resulted, and no harm at any rate followed; and in only four cases were the eyes damaged by the treatment.

Abadie, believing fully in the microbic theory as to the causation of sympathetic ophthalmia, recommends that germicides should be injected, at as early a stage as possible, into the sympathising eye. Two drops of a sublimate solution, 1 in 1000, form the best injection. He also recommends cauterisation of the wound in the exciting eye, and similar injections of sublimate. This treatment may be of some service after sympathy has distinctly occurred; and under circumstances where the damaged eye possesses useful vision, and the indications for removal are not absolute, it might be tried; but there is danger lest in many cases such attempts on the damaged eye should only hasten the onset of sympathetic inflammation in its fellow, and lest the only safe procedure—viz., removal or evisceration of the exciter—should be unduly delayed.

His experiences are, however, especially interesting in connection with Schoelers', as indicating the safety of injecting two or three drops of an aseptic fluid into the eyeball.

Raehlmann draws a distinct separation between trachoma, in which the granulations are always derived from the *follicles* of the conjunctiva, and the chronic purulent conjunctivitis in which the *papillæ* are enlarged. The follicles may be superficial and comparatively innocuous, or they may lie deep in the lid tissues and cause much trouble. The progress of the disease may be acute with profuse discharge, or chronic, in which case there may be no discharge (trachoma siccum), the grey translucent follicles showing in marked contrast to the general hyperæmia of the lids. Where the follicles are numerous and deep-seated, there is much pain from tension, softening, and degeneration of tissue at a later stage, with profuse discharge of pus and of the follicular *débris*. This is followed by chronic thickening of the lid, cicatrisation, and irregular curving of the tarsal cartilage. Pannus seems to depend on the tendency of the corneal tissues, and not only on friction of the lids; but it is more frequent in those cases where the lid-condition is worst. Our author's description of the pathology of the affection would indicate the value of the sharp spoon, or cautery, in clearing out the deep-seated granulation masses and abscesses.

Prince has recently modified an old form of forceps, for the purpose of squeezing out the follicles as they grow; and Arnauts claims good results by instillation of 1 in 120 solution of mercuric bichloride; while Ziebrecht finds the best remedies are one-per-cent. solutions respectively of nitrate of silver or of chloride of zinc.

Dr. Darier of Paris strongly recommends a modification of Sattler's thorough treatment:—(1) Anæsthesia by chloroform. (2) Enlargement of palpebral fissure, by cutting external canthus. (3) Complete exposure of inner surface of lid and cul-de-sac. (4) Deep scarification, so as to let the subconjunctival granulation tissue easily through the conjunctiva without tearing the latter. (5) Erasion of granulations. (6) Brushing with 1 in 500 sublimate solution, which must be thoroughly and carefully done in each recess. (7) Compresses for several days.

Abadie has watched the progress of these cases in his Clinic, and strongly supports this plan of treatment.

It has been suggested by Fränkel that operations upon the eye are especially prone to cause mental derangements. Blindness may be a predisposing cause of low vitality, and conversely cases have been quoted where mental disturbances occurred during the time blindness was coming on, and were cured by the restoration to sight given by the operation. The ill effect of shutting out light by bandaging is strongly stated. The tendency probably depends rather on the mental anxieties associated with loss of vision, than upon the anatomical proximity of the eye to the brain. Senility may be a predisposing factor. Potassium bromide and chloral are recommended when psychoses occur.

Sulzer has studied affections of vision associated with malarial poisoning. During the acute febrile attacks he has described hemorrhage in the neighbourhood of the macula and optic disc; venous hyperæmia of nerve and retina; amblyopia without ophthalmoscopic changes—in some cases amounting to amaurosis which lasted several days, but disappeared with use of quinine. Other symptoms depend upon malarial cachexy, hemorrhage in peripheral portions of retina, diffuse infiltration of the vitreous, and chronic optic neuritis. He has also seen sudden incurable blindness, probably depending on cortical embolism or hemorrhage.

Russian influenza has been a prolific source of inflammations in the eye and its appendages.

Four cases of idiopathic tenonitis are described by Fuchs; in two pneumococci were found in cultivations made from the secretion—one case suppurated.

Hill Griffith quotes Steower's report of a case of proptosis with paralysis of the levator palpebræ and superior rectus due to a similar cause; and two cases of optic atrophy with marked and permanent sight deterioration, for which influenza was the only explicable cause.

Landolt considers that a special form of conjunctivitis was common during convalescence from influenza; it affected the connective tissue of both ocular and palpebral conjunctiva. He also saw several cases of acute œdema of the lids.

Fage (Bordeaux) reports a list of eye-disorders after influenza. He found no connection between the severity of the influenza and the ocular trouble. The latter was usually found somewhat late in the desease, but might occur at its height, or during its decline.

Professor Ladenburg is stated to have tested duboisin sulphate, and to have found it identical with hyoscyamine; subsequently, that he found a sample of duboisin identical with hyoscine, and that in this no hyoscyamine was present. Messrs. Schering state that they worked Duboisia Myoporoides leaves for duboisin, and obtained a base the sulphate of which exactly corresponded to sulphate of hyoscyamine. It seems probable, therefore, that Duboisia Myoporoides contains both hyoscyamine and hyoscine, the product sold as duboisin consisting of the one or the other base, either exclusively or in a preponderance, according to the method of preparation.

Randall recommends as a mydriatic for refraction purposes hyoscyamine sulphate, gr. ij., ad. ℥j., at night, and in the morning before examination is made. He says its action is more prompt and energetic than that of atropia in stronger solutions, and its full action is obtained in about half an hour, and continues for three days; after which, its effect gradually ceases in about two days. He advises, as a rule, full correction in myopia (as well as in hypermetropia); the glass given should accord with the static refraction of the eye, as determined under mydriatic paralysis.

Kolinski points out that naphthalin tends to degeneration of the blood-vessels. The eye being one of the most highly vascular organs, easily shows changes induced by interference with the nutrition of the blood. Naphthalin is said to cause extravasations in the choroid, ecchymoses in the retina, and afterwards cloudiness of the lens and vitreous body.

A few drops of chloroform put into the ear are stated to relieve the photophobia and sneezing which accompany acute keratitis when the light falls upon the cornea. The result is considered by Guttierez-Ponce to be due to production of anæsthesia of the Gasserien ganglion.

Eversbuch has reported a case of ciliary neuralgia, caused by the point of a needle penetrating the eyeball at the sclero-corneal junction; possibly it had detached the iris from its insertion. The symptoms were removed by application of the galvano-cautery to the scar of the original wound.

In the Ophthalmic Hospital at Stuttgart, Koch's fluid has been injected in cases of ulceration of the cornea and in blepharitis of strumous children, with much the same general and local reaction as follow its results in lupus; there was no reaction in two cases of syphilitic iritis, or in one of granular conjunctivitis.

Albrand had a typical result in a patient who suffered from tubercular nodules at the edges of the lids with implication of the associated glands. The treatment seems likely to be of value in very obstinate cases where the disease is entirely extra-ocular.

F. R. Cross.

[*The reports of cases of Koch's treatment, promised in the last number, are deferred until they are completed.*—Ed.]

